# Primary non-response to antiviral therapy affects the prognosis of hepatitis B virus-related hepatocellular carcinoma

**DOI:** 10.1186/s12885-023-11059-y

**Published:** 2023-06-20

**Authors:** Peng Wang, Xinhui Wang, Xiaoli Liu, Fengna Yan, Huiwen Yan, Dongdong Zhou, Lihua Yu, Xianbo Wang, Zhiyun Yang

**Affiliations:** Center of Integrative Medicine, Beijing Ditan Hospital, Capital Medical University, No. 8 Jing Shun East Street, Beijing, 100015 P.R. China

**Keywords:** Hepatocellular carcinoma, Antiviral treatment, Primary non-response, Hepatitis B virus

## Abstract

**Background and aim:**

Although antiviral treatments have been shown to affect the recurrence and long-term survival of patients with hepatocellular carcinoma (HCC) who have high viral loads, the effect of different responses to antiviral therapy on the clinical outcomes remains unclear. This study aimed to assess the effect of primary non-response (no-PR) to antiviral therapy on the survival or prognosis of patients with HCC with a high load of hepatitis B virus (HBV) DNA.

**Methods:**

A total of 493 HBV-HCC patients hospitalized at Beijing Ditan Hospital of Capital Medical University were admitted to this retrospective study. Patients were divided into two groups based on viral response (no-PR and primary response). Kaplan–Meier (KM) curves were used to compare the overall survival of the two cohorts. Serum viral load comparison and subgroup analysis were performed. Additionally, risk factors were screened and the risk score chart was created.

**Results:**

This study consisted of 101 patients with no-PR and 392 patients with primary response. In the different categories based on hepatitis B e antigen and HBV DNA, no-PR group had a poor 1-year overall survival (OS). In addition, in the alanine aminotransferase < 50 IU/L and cirrhosis groups, primary nonresponse was related to poor overall survival and progression-free survival. Based on multivariate risk analysis, primary non-response (hazard ratio (HR) = 1.883, 95% CI 1.289–2.751, *P* = 0.001), tumor multiplicity (HR = 1.488, 95% CI 1.036–2.136, *P* = 0.031), portal vein tumor thrombus (HR = 2.732, 95% CI 1.859–4.015, *P* < 0.001), hemoglobin < 120 g/L (HR = 2.211, 95% CI 1.548–3.158, *P* < 0.001) and tumor size ≥ 5 cm (HR = 2.202, 95% CI 1.533–3.163, *P* < 0.001) were independent risk factors for 1-year OS. According to the scoring chart, patients were divided into three risk groups (high-, medium-, and low-risk groups) with mortality rates of 61.7%, 30.5%, and 14.1%, respectively.

**Conclusions:**

The level of viral decline at 3 months post-antiviral treatment may predict the OS of patients with HBV-related HCC, and primary non-response may shorten the median survival time of patients with high HBV-DNA levels.

**Supplementary Information:**

The online version contains supplementary material available at 10.1186/s12885-023-11059-y.

## Introduction

Liver cancer is the seventh most common cancer and third leading cause of cancer-related deaths worldwide [1]. Hepatocellular carcinoma (HCC) accounts for approximately 90% of primary liver cancers and poses a global health challenge [2]. Hepatitis B virus (HBV) infection can lead to the development and progression of HCC and seriously affect a patient's quality of life [3, 4]. Approximately 80% of HCC cases in China are associated with HBV infection [5]. Currently, surgical resection (SR) is considered the most appropriate treatment among various clinical treatments for HCC [6–8]. However, Recurrence of HCC is an important factor that adversely affects postoperative survival [9]. In addition to surgical factors, other factors such as hepatitis viral load and the degree of cirrhosis have also been confirmed to be risk factors for liver cancer recurrence [10]. Therefore, the long-term prognosis of HCC remains poor. In addition to curative therapies, adjuvant therapies that prolong survival or prevent recurrence are urgently needed for patients with HCC.

Some studies have reported that a high viral load affects the prognosis of patients with HCC, and decrease of HBV DNA to low or undetectable levels is considered to be a desirable endpoint for liver cancer treatment [11–13]. Antiviral therapy with oral nucleotide analogs (NAs) has been found to prevent tumor progression, reduce recurrence, and prolong overall survival (OS) in patients with HCC after hepatectomy [14–17]. Increasing evidence suggests that antiviral treatment is associated with HBV-HCC prognosis and comprehensive treatment outcomes [18]. Therefore, antiviral therapies may be effective for the tertiary prevention of HBV-HCC. However, among patients with liver cancer who undergo antiviral therapy, a poor virological response, although rare, still exists. Therefore, in clinical practice, specific indicators are needed to evaluate the effectiveness of antiviral therapy for HCC, including effective indicators and time needed to achieve these indicators.

A previous retrospective study suggested that different viral responses are related to death or HCC in a cirrhotic population [19]. Other studies have reported that primary non-response in patients with chronic hepatitis B (CHB) infection may lead to failure of antiviral therapy and progression of liver fibrosis [20, 21] However, whether different levels of viral response affect the clinical outcomes in HBV-HCC remains uncertain. Therefore, it is necessary to evaluate HBV-HCC with poor antiviral effects through further studies. Based on the findings, the treatment strategy could then be adjusted over time. This study aimed to assess the effect of primary non-response to short-term antiviral therapy on the outcome or prognosis of patients with HCC with high levels of HBV DNA.

## Methods

### Patients

We retrospectively enrolled 742 patients with HBV-related HCC at Beijing Ditan Hospital affiliated to Capital Medical University between December 2008 and September 2015 whose HBV-DNA levels were ≥ 2000 IU/mL. This study was approved by the ethics committee of Beijing Ditan Hospital. Inclusion criteria for this study were: (1) patients were diagnosed with primary liver cancer; (2) serum HBV DNA ≥ 2000 IU/mL and without nucleos(t)ide (NUC) analog treatment for at least 6 months; and (3) aged 18–75 years. Exclusion criteria included: (1) tumor caused by other factors, such as hepatitis C virus (HCV), hepatitis D virus (HDV), or alcohol (*n* = 47); (2) incomplete data (*n* = 29); (3) less than three months of follow-up (*n* = 121); and (4) absence of HBV DNA after 3 months (*n* = 52).

HBV-HCC patients were defined by histopathological or clinical diagnosis using at least two imaging methods that showed clear lesions (hepatic angiography, magnetic resonance imaging, or liver ultrasonography) or through one display imaging method showing a clear lesion plus alpha-fetoprotein (AFP) ≥ 400 ng/ml. Tumor size was defined as the diameter of the largest tumor. Cirrhosis was defined based on histological or ultrasonographic findings. Liver stiffness measurement was assessed by transient elastography (TE) using FibroScan® (Echosens, Paris, France). HBV treatment mainly included long-term oral NUCs for at least 12 months. HBV-DNA levels were recorded at baseline and every 3–6 months during the follow-up. All patients received adequate treatment, and the indications for NA therapy and antitumor treatment followed the guidelines of the American Association for the Study of Liver Diseases (AASLD) and the European Association for the Study of the Liver (EASL) [3, 22]. After initial therapy, patients eligible for post-treatment receive SR, TACE, RFA, and/or sorafenib as needed. Post-treatment depends on liver function, tumor burden and patient requirements. After 6–8 weeks of treatment, repeat TACE or RFA in combination with sorafenib are recommended if residual tumor enhancement and vascularity can be observed on imaging. Sorafenib therapy is recommended for patients who are not suitable for any post-treatment. As liver transplantation was not performed at our hospital, liver transplantation was not considered as post-treatment for any patients. Disease-related deaths were defined as follows: (1) tumor-related deaths, which mainly result from tumor recurrence or complications during treatment; (2) liver-related deaths, which were caused by advanced HCC complications, including gastroesophageal variceal bleeding.

### Definitions

A decrease of < 1 log10 of serum HBV DNA after 3 months of therapy was defined as a primary non-response (no-PR) [22]. Otherwise, it was defined as primary response (PR). HBV-HCC refers to patients whose serum is positive for hepatitis B surface antigen (HBsAg; ≥ 6 months) and who meet the diagnostic criteria for HCC. OS was defined as the time from randomization to death due to any cause. Progress-free survival (PFS) was defined as the time from randomization to the first recorded disease progression. Treatment were based on guidelines of the AASLD and EASL when HBV-DNA levels were high (> 2000 IU/mL) [3, 22].

### Statistical analysis

SPSS 23.0 and GraphPad 8.0 were used for data analysis. Quantitative data conforming to a normal distribution are expressed as mean ± standard deviation (SD). A *t*-test was used to compare the means of two groups. Non-normally distributed data are expressed as median and quartile ranges (M, QR). Comparisons between two groups were performed using the Mann–Whitney U test. Qualitative data are expressed as frequency, and the comparison between two groups was performed using the χ^2^ test. Differences in survival among PR and no-PR patients in different groups were also assessed using the Kaplan–Meier (KM) curve and log-rank test. 1:3 Propensity score matching (PSM) was used to reduce confounding factors between the PR and NOPR groups. Cox regression analysis was used to elucidate independent risk factors affecting the 1-year OS. Statistical significance was set at *P* < 0.05.

## Results

### Baseline characteristics

**Fig. 1:**
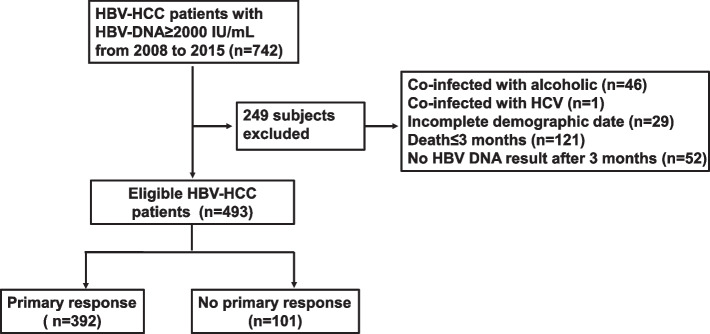
Flow chart showing the patients included in the study. HCC, hepatocellular carcinoma; HBV, hepatitis B virus; HCV, hepatitis C virus

Based on the inclusion and exclusion criteria, 493 patients were screened and a flow chart with enrollment details is shown in Fig. 1. The study cohort consisted of 387 men and 106 women, with a median age of 55.31. The baseline characteristics of the patients are summarized in Table 1. A total of 392 patients formed the PR group and 101 patients formed the no-PR group. The proportion of portal vein tumor thrombus (PVTT) in the PR group was lower than that in the no-PR group (*P* < 0.05). Markedly higher levels of alanine aminotransferase (ALT), HBV DNA, or hemoglobin (HGB) were observed in the PR group than in the no-PR group. However, patients in the PR group exhibited lower total bilirubin (TBiL) levels. In the no-PR group, more patients received systemic therapy (34.7% vs 15.6%, *p* < 0.001) and few patients received transarterial chemoembolization combined with radiofrequency ablation (TACE + RFA; 41.3% vs 26.7%, *p* = 0.007). The proportion of extrahepatic metastasis and previous antiviral treatment were not significantly different between the two cohorts (*P* > 0.05).

**Table 1:** Demographic data and clinical characteristics of the patients

	Total(*n* = 493)	PR(*n* = 392)	No-PR(*n* = 101)	*P* value
Sex				0.846
Male	387(78.5)	307(78.3)	80(79.2)	
Female	106(21.5)	85(21.7)	21(20.8)	
Age	55.31 ± 9.91	55.38 ± 9.89	55.07 ± 10.05	0.866
Smoking	204(41.4)	169(43.1)	35(34.7)	0.124
Alcohol	166(33.7)	140(35.7)	26(25.7)	0.059
Cirrhosis	456(92.5)	362(92.3)	94(93.1)	0.806
Hypertension	121(24.5)	102(26.0)	19(18.8)	0.133
Child Staging				0.100
A	234(47.5)	195(49.7)	39(38.6)	
B	177(35.9)	137(34.9)	40(39.6)	
C	82(16.6)	60(15.3)	22(21.8)	
HBV-DNA (log10IU/L)	4.6 ± 1.15	4.8 ± 1.13	4.1 ± 1.08	< 0.001
HBeAg at basline				0.421
Negative	247(50.1)	200(51)	47(46.5)	
Positive	246(50.9)	192(49)	54(53.5)	
ALT (U/L)	44.2(30.45,68.85)	47.8(32.0,73.23)	34.3(25.75,50.25)	< 0.001
TBIL (g/L)	20.3(13.7,32.8)	19.8(13.23,31.68)	22.6(14.65,38.65)	0.042
PLT (10^9^/L)	88.3(57.3,138.95)	90.35(59.05,138.3)	79.4(50.25,140.4)	0.204
HGB (g/L)	126.4(109.8,140.6)	127.7(112.7,140.9)	120.9(102.0,137.5)	0.047
AFP (ng/ml)	39.6(8.83,210.08)	39.95(8.63,214.53)	39.15(10.2,207.05)	0.895
Tumor multiplicity				0.951
Solitary	272 (55.2)	216 (55.1)	56 (55.4)	
Multiple	221 (44.8)	176 (44.9)	45 (44.6)	
Tumor size				0.106
≤ 5 cm	354 (71.8)	288 (73.5)	66 (65.3)	
> 5 cm	139 (28.2)	104 (26.5)	35 (34.7)	
PVTT	88 (17.8)	63 (16.1)	25 (24.8)	0.042
Extrahepatic metastasis	93 (18.8)	71 (18.1)	22 (21.8)	0.401
BCLC				
0-A	161 (32.7)	134 (34.2)	27 (26.7)	0.154
B	177 (35.9)	142 (36.2)	35 (34.7)	0.769
C	73 (14.8)	56 (14.3)	17 (16.8)	0.521
D	82 (16.6)	60 (15.3)	22 (21.8)	0.119
MELD	5.6 (2.6,8.83)	5.48 (2.39,8.43)	5.96 (3.57,10.19)	0.051
Previous antiviral treatment	86 (17.4)	63 (16.1)	23 (22.8)	0.114
Treatment for HCC				
Surgery	41 (8.3)	35 (8.9)	6 (5.9)	0.169
Systemic therapy	96 (19.5)	61 (15.6)	35 (34.7)	< 0.001
TACE	146 (29.6)	116 (29.6)	30 (29.7)	0.983
RFA	21 (4.3)	18 (4.6)	3 (3.0)	0.784
TACE + RFA	189 (38.3)	162 (41.3)	27 (26.7)	0.007

*ALT* Alanine aminotransferase, *TBIL* Total bilirubin, *PLT* Platelets, *HGB* Hemoglobin, *AFP* α-fetoprotein, *PVTT* Portal vein tumor thrombus, *BCLC* Barcelona Clinic Liver Cancer staging system, *HBeAg* Hepatitis B e antigen, *HBV* Hepatitis B virus, *MELD* Model for end-stage liver disease, *TACE* Transarterial chemoembolization, *RFA* Radiofrequency ablation

### Viral response after three months of NA therapy

**Fig. 2:**
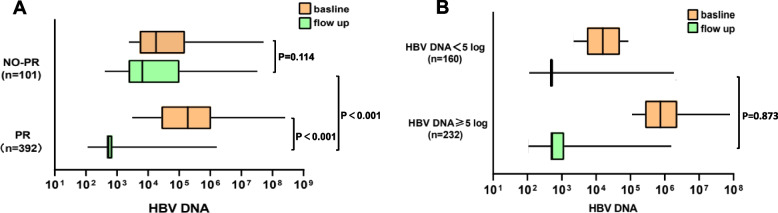
Baseline and follow-up HBV DNA level. The box plots show the median (vertical bar), 25th and 75th percentiles. **A** Primary response(PR) and primary non-response(no-PR). **B** Higher viral load(HBV DNA ≥ 5log) and lower viral load(HBV DNA < 5log) groups in PR

A total of 493 cases with HBV DNA data (HBV DNA values after three months of antiviral treatment) were available for analysis. Of the 493 patients, 407 were naive patients: including 264 patients treated with entecavir, 52 patients treated with lamivudine, 83 patients treated with adefovir dipivoxil, and eight patients treated with telbivudine. The viral load in patients receiving NA treatment is shown in Fig. 2A-B. As shown in Fig. 2A, HBV-DNA levels in the no-PR group were higher than those in the PR group after three months of treatment (*p* < 0.001). Based on the baseline viral levels, we assigned the PR patients to two groups: a higher-level group (HBV DNA ≥ 5log) and a lower-level group (HBV DNA < 5log). There was no statistically significant difference between the two subgroups of PR patients (Fig. 2B).

### Survival analysis of all patients before and after PSM processing

**Fig. 3:**
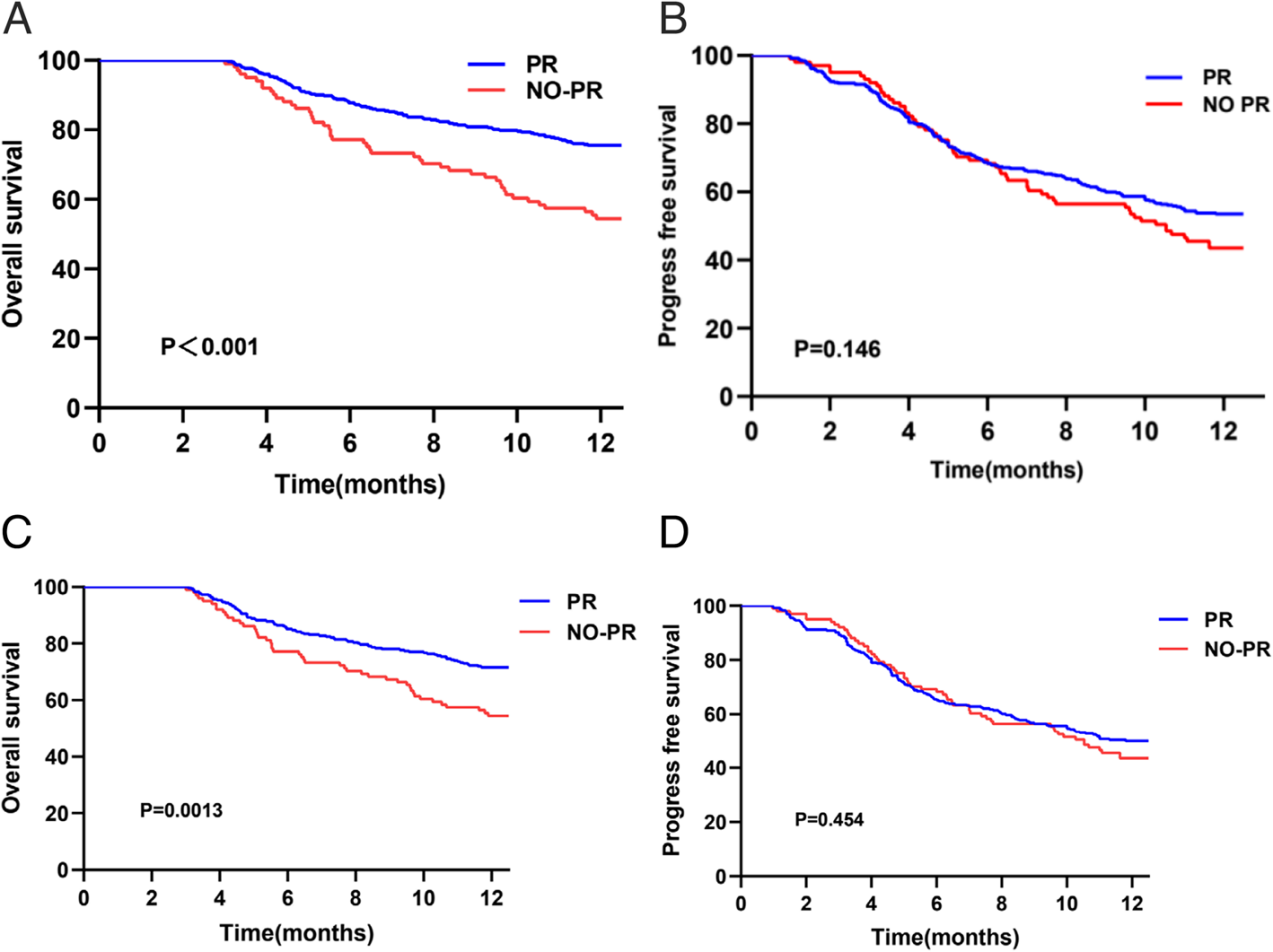
Kaplan–Meier curve analysis showing overall survival (OS) and progression-free survival (PFS) in two groups before and after propensity score matching (PSM). **A** 1-year OS. **B** 1-year PFS. **C** 1-year OS after PSM. **D** 1-year PFS after PSM

All patients were followed-up from the time of first hospitalization. The median follow-up time for the entire study population was 34.5 months. The median survival times of the PR and no-PR groups were 39.6 and 15 months, respectively. Among the 142 (28.8%) patient deaths that occurred within a year, 93 (18.9%) were tumor-related deaths, including 59 (15%) in the PR group and 34 (33%) in the no-PR group. In addition, 49 (10%) were liver-related deaths, including 37 (9%) cases in the PR group and 12 (12%) in the no-PR group. The 1-year OS rate was 75.5% in the PR group and 54.5% in the no-PR group. K-M analysis indicated that the PR group had a better cumulative survival rate (log-rank *p* < 0.001) (Fig. 3A). However, 1-year progression-free survival (PFS) was not significantly different between the two cohorts (*P* > 0.05; Fig. 3B). In order to exclude the effect of confounding factors, logistic regression was used for 1:3 PSM to ensure that the two groups could be fairly compared (Supplementary Table 1). Propensity score analysis mainly included demographic or clinical characteristics such as age, gender, PVTT, HGB, tumor size, and number of tumors at baseline. Similarly, PR group had a significantly higher 1-year overall survival rate than the no-PR group (log-rank *p* = 0.0013) (Fig. 3C). PFS was not significantly different between the two groups (Fig. 3D).

**Fig. 4:**
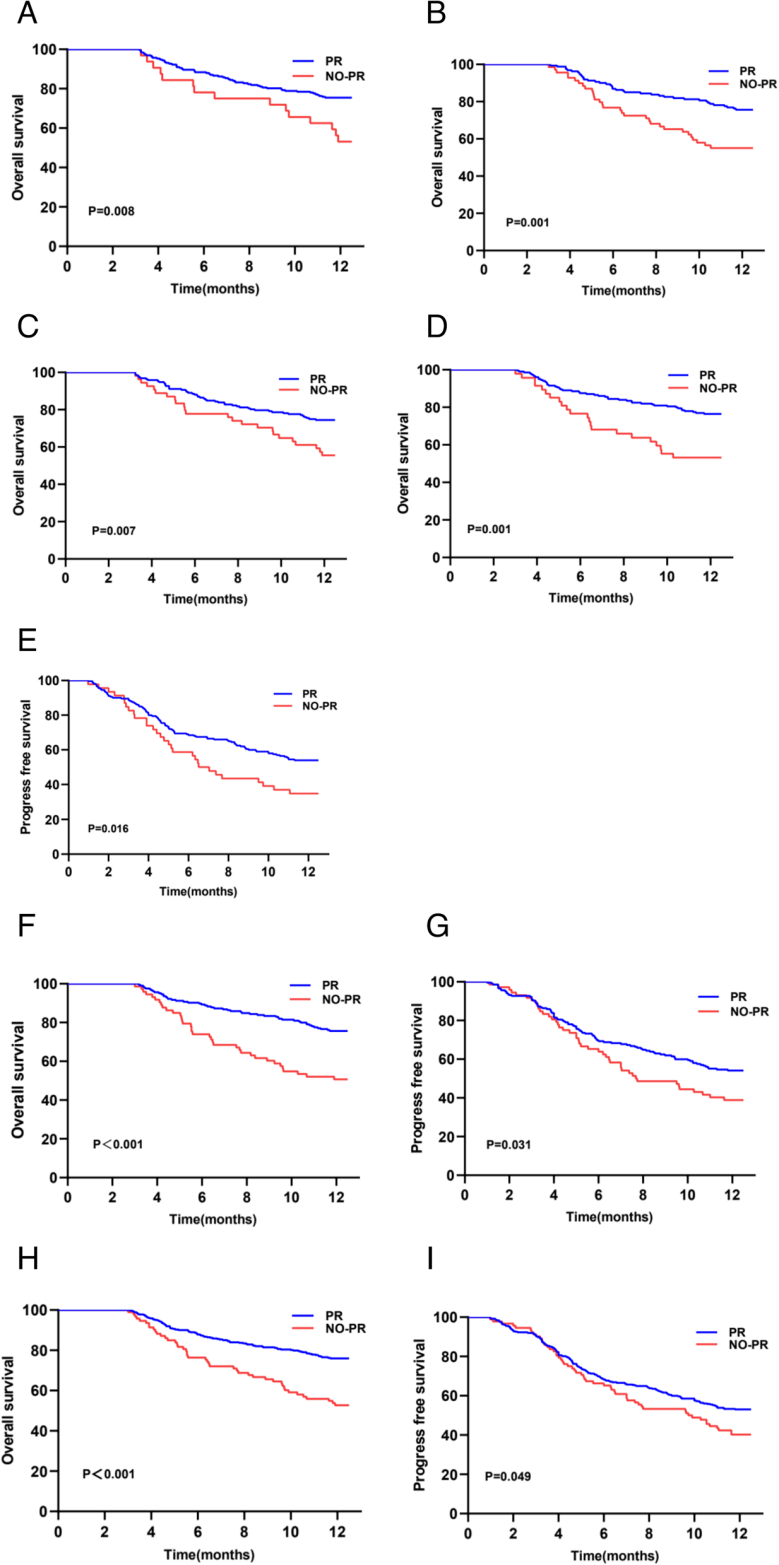
Kaplan–Meier curve analysis showing 1-year overall survival (OS) and progression-free survival (PFS) in two groups. **A**, **B** OS in higher viral load(HBV DNA ≥ 5log) and lower viral load(HBV DNA < 5log) groups. **C**, **D** OS of HBeAg( +) and HBeAg(-) patients. **E** PFS of HBeAg(-) patients. **F**, **G** OS and PFS of patients with ALT < 50U/L. **H**, **I** OS and PFS of patients with cirrhosis

Furthermore, the effect of different viral responses on 1-year OS and 1-year PFS were analyzed in the different categories of etiologies (cirrhosis or no cirrhosis), HBV DNA (high or low), serum ALT (> 50 U/L or ≤ 50 U/L) level, and HBeAg (positive or negative) status. The results of subgroup analysis suggested that PR patients had a significantly higher survival rate than no-PR patients regardless of their HBV-DNA level and HBeAg status (Fig. 4A-D). PFS was distinctly different only in HBeAg-negative patients (Fig. 4E). PR patients with alanine aminotransferase (ALT) < 50 IU/L and cirrhosis showed higher OS and PFS (Fig. 4F-I).

**Fig. 5:**
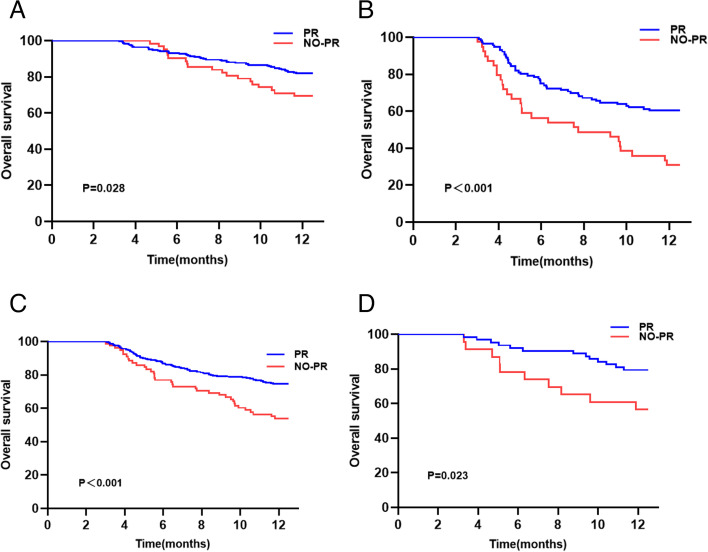
Kaplan–Meier curve showing 1-year overall survival (OS) in the two groups. **A**, **B** OS in BCLC 0-B and **C**, **D** groups. **C**, **D** OS in naïve and no-naïve groups

We then evaluated the effect of each Barcelona Clinic Liver Cancer (BCLC) stage, grouped based on OS (Fig. 5). The results showed that in BCLC 0-B or C-D, the PR group had a higher cumulative survival rate. As shown in Fig. 5C and D, there were statistically significant differences in both groups of patients, regardless of whether they were naïve. We also compared the different treatments between the PR and no-PR groups (Supplementary Fig. 1A-E). The 1-year OS between the two groups was statistically different only in patients who underwent TACE, and the no-PR group had a significantly lower OS rate (*p* = 0.004). Similarly, subgroup analysis showed that NOPR was a risk factor in the BCLC 0-B, BCLC C-D, and minimally invasive groups (Supplementary Fig. 2).

### Univariate and multivariate analysis

Latent variables for predicting 1-year mortality were evaluated using univariate analysis with the log-rank test. Tumor size, tumor multiplicity, ALT, TBiL, red blood cell (RBC), creatinine (CR), No-PR, HGB, Child–Pugh score, BCLC, and other closely related variables in the univariate analysis were included in the multi-factor Cox regression analysis. HGB < 120 g/L (HR = 2.211, 95% CI 1.548–3.158, *P* < 0.001), no-PR( HR = 1.883, 95% CI 1.289–2.751, *P* = 0.001), PVTT at baseline (HR = 2.732, 95% CI 1.859–4.015, *P* < 0.001), tumor size ≥ 5 cm ( HR = 2.202, 95% CI 1.533–3.163, *P* < 0.001), and tumor multiplicity (HR = 1.488, 95% CI 1.036–2.136, *P* = 0.031) were identified as independent risk factors for 1-year mortality by multivariate analysis (Table 2).

**Table 2:** Univariate and multivariate analyses of risk factors associated with 1-year mortality in the patients

	Univariate			Multivariate		
	HR	95%CI	*P* value	HR	95%CI	*P* value
Sex	1.154	0.763–1.745	0.496			
Age (≥ 50)	0.817	0.570–1.171	0.271			
Alcohol	1.140	0.809–1.607	0.453			
Tumor size (≥ 5 cm)	2.576	1.816–3.656	< 0.001	2.202	1.533–3.163	< 0.001
Tumor multiplicity	2.009	1.438–2.805	< 0.001	1.488	1.036–2.136	0.031
WBC (10^9^/L)	1.193	0.899–1.582	0.222			
ALT (U/L)	0.821	0.586–1.149	0.250			
PLT (10^9^/L)	1.145	0.823–1.592	0.421			
TBIL (μmol/L)	1.293	0.925–1.807	0.132			
RBC	0.465	0.326–0.664	< 0.001			
Cr	3.416	1.737–6.718	< 0.001			
AFP > 400 ng/ml	1.309	0.799–2.144	0.285			
no-PR	2.119	1.491–3.013	< 0.001	1.883	1.289–2.751	0.001
HGB (g/L)	2.358	1.694–3.283	< 0.001	2.211	1.548–3.158	< 0.001
Child (A-B)	0.530	0.362–0.777	0.001			
HBeAg ( +)	1.060	0.763–1.473	0.728			
BCLC (C-D)	2.939	2.113–4.087	< 0.001			
Cirrhosis	0.999	0.540–1.848	0.997			
PVTT at baseline	4.011	2.841–5.662	< 0.001	2.732	1.859–4.015	< 0.001
Treatment (TACE)	1.412	1.004–1.987	0.048			

*WBC* White blood cell, *ALT* Alanine aminotransferase, *PLT* Platelet, *TBIL* Total bilirubin, *Cr* Creatinine, *RBC* Red blood cell, *HGB* Hemoglobin, *AFP* α-fetoprotein, *NO-PR* Primary non response, *PVTT* Portal vein tumor thrombus, *BCLC* Barcelona Clinic Liver Cancer staging system, *HBeAg* Hepatitis B e antigen

### Risk score chart

**Fig. 6:**
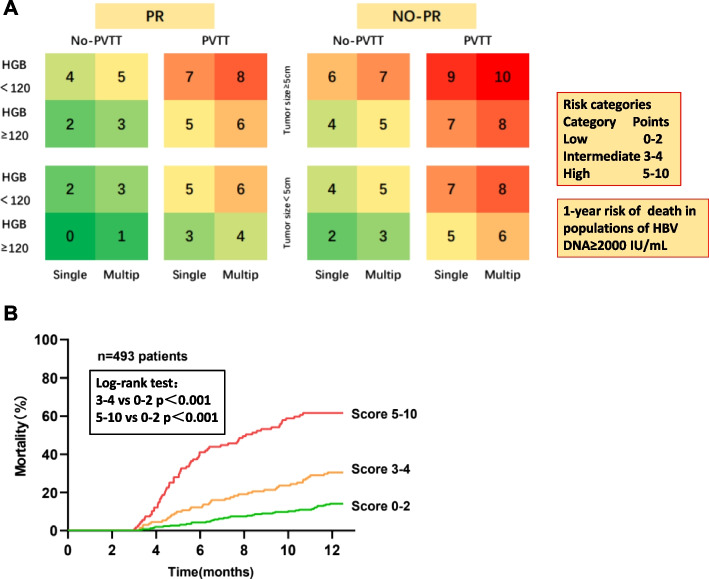
**A** One-year risk of death in populations with HBV DNA ≥ 2000 IU/L. Total score is based on risk categories: low: 0–2 points; intermediate: 3–4 points; and high: 5–10 points. **B** Kaplan–Meier curve showing 1-year mortality in different risk categories

Risk score chart was created based on multivariable analysis and contained the following five factors that retained statistical significance: HGB < 120 g/L, no-PR, tumor size ≥ 5 cm, PVTT, and tumor multiplicity. Subsequently, risk score was created by assigning 1, 2, or 3 points (Rounding the parameters with HR ≥ 2.5 as 3 points, 1.5 ≤ HR < 2 as 2 points, and HR < 1.5 as 1 point) to the variables (Table 3). The score ranged from 0 to 10 points. Using this score, the population was divided into three risk categories: low (0–2 points), medium (3–4 points), and high (5–10 points). Score risk charts showed the 1-year risk of death for 32 combinations of risk factors for the high-, intermediate-, and low-risk regions, as shown in Fig. 6A. Of the 493 patients, 255 (51.7%) were in low, 131 (26.6%) in intermediate, and 107 (21.7%) in high-risk categories.

**Table 3:** Risk score of mortality

Variable	HR	*P* value	Points
no-PR	1.883	0.001	2
HGB	2.211	< 0.001	2
PVTT	2.732	< 0.001	3
Tumor size ≥ 5 cm	2.202	< 0.001	2
Tumor multiplicity	1.488	0.031	1
Maximum			10

Using stepwise multivariable regression analysis, we created a risk score chart for mortality in patients with HCC. The scoring system was determined by rounding the respective parameter estimates and assigning 1, 2, or 3 points to each variable based on the observed hazard ratio

In the low-, intermediate-, and high-risk categories, the mortality rates calculated using the chi-square test were 14.1%, 30.5%, and 61.7%, respectively (*p* < 0.001). Figure 6B shows the Kaplan–Meier curves for 1-year mortality in the three score categories.

## Discussion

High HBV viral load is a prognostic risk factor for liver cancer [11, 12]. In HBV-related HCC, the sooner the antiviral therapy helps achieve undetectable HBV-DNA levels, the better the prognosis of the patient [13]. Antiviral treatments provide significant benefits in reducing recurrence and improving the survival rate of patients with HBV-related HCC after surgical resection [16, 23, 24]. A previous study revealed that antiviral therapy was effective in reducing the postoperative recurrence of liver cancer in patients with high HBV-DNA levels and a predictor of long-term survival [25]. We found that in a small number of HCC patients the viral load decreased slowly after short-term antiviral treatment. It is not yet clear whether this subset of patients has a different outcome than that of those who respond well. A study suggested that patients with decompensated cirrhosis with good viral response have a lower likelihood of developing HCC after 12-months of entecavir treatment [26]. In addition, primary non-response has been found to lead to the failure of antiviral therapy and progression of CHB infection [20, 21]. This study is the first to analyze the effect of different viral responses after short-term antiviral therapy on the prognosis of HCC patients. The results suggest that primary non-response is a risk factor for survival of HBV-HCC patients.

We found that the average baseline level of HBV DNA in the PR group was higher than that in the no-PR group. However, after three months of NA treatment, the former had a lower average HBV-DNA level. We further divided the PR patients into two subgroups based on their baseline levels as higher levels (HBV-DNA ≥ 5log^10^) and relatively low levels (3log^10^ ≤ HBV-DNA < 5log^10^) groups, and evaluated whether low DNA level after 3 months was associated with high baseline DNA levels. The average DNA levels in the two populations were not statistically significant after 3 months (*p* = 0.873). According to recent studies, low-level viremia affects the virological response to subsequent treatment [27]and was associated with worse clinical outcomes in compensated cirrhosis patients who were not receiving antiviral treatment [28]. It is more difficult to reduce HBV DNA in patients with low-level viremia; therefore, a stronger antiviral treatment strategy is necessary in the clinic. However, in this study, we found that the baseline HBV-DNA levels in the primary non-response group were lower. However, as shown in Fig. 2B, a lower baseline level does not affect whether patients with HBV-HCC develop a primary non-response. In other words, patients with HBV-related HCC should continue to monitor their viral levels, regardless of their baseline levels. If the antiviral treatment is ineffective, then the antiviral strategy should be changed in time to avoid primary non-response, thereby improving the prognosis of the patient.

In this study, the PR group had a higher 1-year survival rate than the no-PR group. However, no significant difference was found between the two groups in the 1-year PFS. Subsequently, we analyzed the effect of no-PR in all the HCC-related subgroups. Related studies have reported tumor recurrence accompanied by liver decompensation as one of the main causes of death during follow-up [29, 30]. Our results showed that patients with PR in the cirrhosis group had better OS and PFS than those without PR in the same stage, but not in those without cirrhosis. This may be related to the reversal of the cirrhosis. Antiviral therapies mediate their effect mainly by inhibiting liver inflammation, preventing HBV reactivation, and reversing liver fibrosis [31]. Furthermore, regardless of HBV-DNA levels or HBeAg status at baseline, PR patients have higher survival rates than no-PR patients. The underlying mechanism is unclear and may be related to the inhibition of HBV activation and improvement of liver function. The absence of PR decreased the chances of subsequent curative treatments, thereby decreasing the OS of patients with HCC. HBV replication induces cancer through both direct and indirect carcinogenic mechanisms [32, 33]. Compared to patients who achieve viral response within the first 12 months, some studies suggest that patients with residual viremia during therapy may have a higher risk of developing HCC [34, 35]. The existence of viremia is believed to weaken the immune monitoring of tumors leading to the development of multi-center carcinogenesis within the liver residues in patients with viral residues, and upregulate molecules of hepatic sinus intima cells, thereby promoting tumor spread [36, 37]. The present study found that the proportion of patients with undetectable HBV-DNA levels in the PR group was significantly higher than that in the no-PR group during follow-up. In addition, we observed that the PFS of the two cohorts was statistically significant only in the HBeAg-negative (*P* = 0.016), normal ALT (*P* = 0.031), and cirrhosis (*P* = 0.049) populations. This may be due to the high recurrence rate of liver cancer [9]. In addition, the impact of short-term antiviral therapy on PFS may be limited. However, further research is needed to confirm this.

The independent risk factors for 1-year OS in HBV-related HCC with high HBV-DNA levels were HGB level < 120 g/L, no-PR, PVTT, tumor size ≥ 5 cm, and tumor multiplicity. Known independent prognostic factors for death in HBV-related HCC include PVTT, tumor size, tumor number, HBV DNA, AST, and Child–Pugh score [38, 39]. Previous studies have also demonstrated that patients with anemia have increased risk of death compared to those without anemia [40, 41]. The PVTT, HGB, tumor size, and tumor multiplicity data in this study correlated with those of previous studies, and no-PR after 3 months was newly identified as a predictive factor in the present study.

Furthermore, we established a risk-scoring system based on no-PR and four other factors to predict the mortality risk in HCC during the follow-up period. The risk score charts showed 32 different combinations, and the score for each combination is clearly displayed. The results of our analysis show that based on the different score categories, mortality rate exhibits a gradually increasing trend. In addition, the score showed good performance in predicting the 1-year mortality risk in all study cohorts. Therefore, we believe that this study provides valuable clinical information. However, the score risk charts may be used for a rough assessment of mortality risk in HCC patients with high HBV-DNA levels.

This study has several limitations. First, it was a retrospective cohort study and therefore some of the data and clinical characteristics in the two groups may have been inevitably biased. Second, owing to equipment limitations at the time, HBV-DNA levels below 500 IU/mL could not be detected. However, with the latest standards, undetectable HBV-DNA levels have dropped below 20 IU/mL, so that we can now analyze the virus response more clearly. Third, owing to the lower baseline level of HBV DNA in the no-PR group, the impact of NA treatment on OS may be underestimated. Fourth, since PFS is a composite endpoint, a competing risk effect of death due to the natural history of cirrhosis is present in addition to the association with tumor progression [3]. Deaths due to liver dysfunction such as gastrointestinal bleeding, infection or hepatic encephalopathy were present in this study. Therefore, deaths unrelated to tumor progression may be the important factor affecting PFS. Fifth, this retrospective study lacked viral data from multiple time points, which may have resulted in changes in the virus that could not be compared. In fact, our study focused on the impact of short-term antiviral changes on patient prognosis, which is one of the innovative aspects of this study. Based on our results, we still believe that short-term viral response remains one of the important factors of prognosis regardless of the longer-term changes. Finally, the sample size was small and further studies with more patients are needed to confirm the data.

In conclusion, we found that the levels of viral decline at 3 months can predict 1-year OS, and primary non-response at 3 months potentially shortens the median survival time among patients with HCC with high HBV-DNA levels. Therefore, antiviral programs for HBV-HCC patients may need to be adjusted when the decline in viral load is less than 1 log in three months.

## Supplementary Information

**Additional file 1: Supplementary Table 1.** Demographic data and clinical characteristics of the patients after the 1:3 PSM. **Supplementary Figure 1.** Kaplan-Meier curve showing 1-year overall survival (OS) in the two groups. **Supplementary Figure 2.** Subgroup analysis according to tumor stage and treatment modality.

## Data Availability

The data used to support the findings of this study are available from the corresponding author upon request. Our study was a retrospective cohort research, which collect the clinical data of patients. The researchers will try their best to protect the information provided by patients from disclosing personal privacy.
