# Supplementary material for: Primary non-response to antiviral therapy affects the prognosis of hepatitis B virus-related hepatocellular carcinoma
Source: BMC Cancer. 2023 Jun 20;23:564. doi: 10.1186/s12885-023-11059-y (PMC10280839; doi:10.1186/s12885-023-11059-y)
Supplement: Supplementary file 1 — Additional file 1: Supplementary Table 1. Demographic data and clinical characteristics of the patients after the 1:3 PSM. Supplementary Figure 1. Kaplan-Meier curve showing 1-year overall survival (OS) in the two groups. Supplementary Figure 2. Subgroup analysis according to tumor stage and treatment modality. [file 12885_2023_11059_MOESM1_ESM.docx]

**Supplementary Table 1.** Demographic data and clinical characteristics of the patients after the 1:3 PSM.

|  | Total（n=397） | PR（n=296） | No-PR（n=101） | P value |
| --- | --- | --- | --- | --- |
| Sex |  |  |  | 0.555 |
| Male | 306（77.1） | 226（76.4） | 80（79.2） |  |
| Female | 91（22.9） | 70（23.6） | 21（20.8） |  |
| Age | 55.72±9.38 | 55.94±9.15 | 55.07±10.05 | 0.365 |
| Smoking | 160（40.3） | 125（42.2） | 35（34.7） | 0.180 |
| Alcohol | 130（32.7） | 104（35.1） | 26（25.7） | 0.082 |
| Cirrhosis | 369（92.9） | 276（93.2） | 93（92.1） | 0.693 |
| Hypertension | 98（24.7） | 79（26.7） | 19（18.8） | 0.113 |
| Child Staging |  |  |  | 0.468 |
| A | 173（43.6） | 134（45.3） | 39（38.6） |  |
| B | 149（37.5） | 109（36.8） | 40（39.6） |  |
| C | 75（18.9） | 53（17.9） | 22（21.8） |  |
| HBV-DNA (log10IU/L) | 4.5±1.12 | 4.7±1.13 | 4.1±1.08 | ＜0.001 |
| HBeAg at basline |  |  |  | 0.284 |
| Negative | 203（51.1） | 156（52.7） | 47（46.5） |  |
| Positive | 194（48.9） | 140（47.3） | 54（53.5） |  |
| ALT (U/L) | 43.7(30.3,64.8) | 46.6(31.2,70.2) | 34.3(25.75,50.25) | 0.002 |
| TBIL (g/L) | 21.2(14.4,33.0) | 20.5(13.8,32.0) | 22.6(14.65,38.65) | 0.100 |
| PLT (10^9^/L) | 88.4(56.7,139.0) | 91.0(57.8,138.3) | 79.4(50.25,140.4) | 0.372 |
| HGB (g/L) | 122.8(107.0,140.5) | 123.6(107.7,140.6) | 120.9(102.0,137.5) | 0.536 |
| AFP (ng/ml) | 32.4(8.1,206.6) | 31.1(7.7,206.6) | 39.15(10.2,207.05) | 0.659 |
| Tumor multiplicity |  |  |  | 0.808 |
| Solitary | 216 (54.4) | 160 (54.1) | 56 (55.4) |  |
| Multiple | 181 (45.6) | 136 (45.9) | 45 (44.6) |  |
| Tumor size |  |  |  | 0.923 |
| ≤5cm | 261 (65.7) | 195 (65.9) | 66 (65.3) |  |
| ＞5cm | 136 (34.3) | 101 (34.1) | 35 (34.7) |  |
| PVTT | 87 (21.9) | 62 (20.9) | 25 (24.8) | 0.699 |
| Extrahepatic metastasis | 81 (20.4) | 59 (19.9) | 22 (21.8) | 0.506 |
| BCLC |  |  |  |  |
| 0-A | 110 (27.7) | 83 (28.0) | 27 (26.7) | 0.615 |
| B | 142 (35.8) | 107 (36.1) | 35 (34.7) | 0.556 |
| C | 70 (17.6) | 53 (17.9) | 17 (16.8) | 0.701 |
| D | 75 (18.9) | 53 (17.9) | 22 (21.8) | 0.186 |
| MELD | 5.7 (2.6,9.1) | 5.59 (2.28,8.67) | 5.96 (3.57,10.19） | 0.471 |
| Previous antiviral treatment | 72 (18.1) | 49 (16.6) | 23 (22.8) | 0.154 |
| Treatment for HCC |  |  |  |  |
| Surgery | 29 (7.3) | 23 (7.8) | 6 (5.9) | 0.121 |
| Systemic therapy | 88 (22.2) | 53 (17.9) | 35 (34.7) | ＜0.001 |
| TACE | 121 (30.5) | 91 (30.7) | 30 (29.7) | 0.956 |
| RFA | 16 (4.0) | 13 (4.4) | 3 (3.0) | 0.402 |
| TACE+RFA | 143 (36.0) | 116 (39.2) | 27 (26.7) | 0.027 |

ALT, alanine aminotransferase; TBIL, total bilirubin; PLT, platelets; HGB, hemoglobin; AFP, α-fetoprotein; PVTT, portal vein tumor thrombus; BCLC, Barcelona Clinic Liver Cancer staging system; HBeAg hepatitis B e antigen; HBV hepatitis B virus; MELD model for end-stage liver disease; TACE, transarterial chemoembolization; RFA, radiofrequency ablation, PSM, Propensity score matching.

**Supplementary Figure 1**

A B


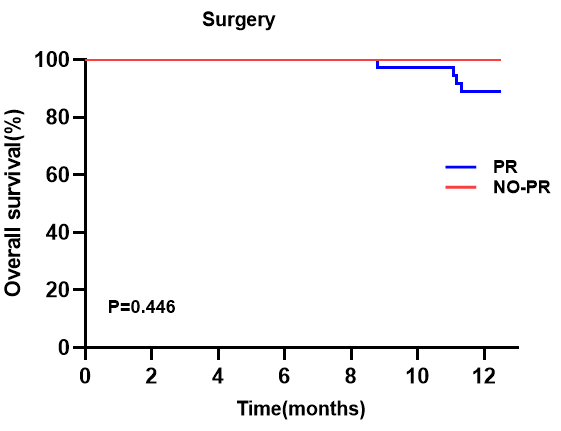

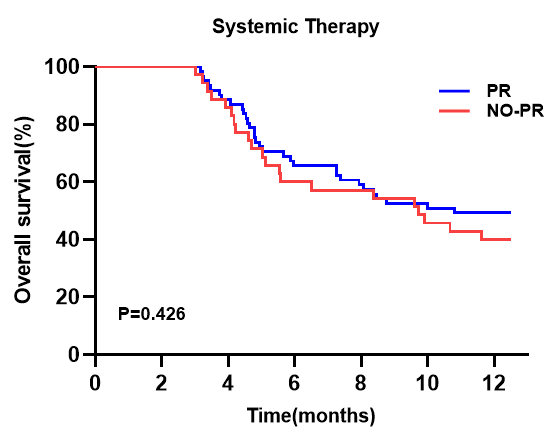


C D


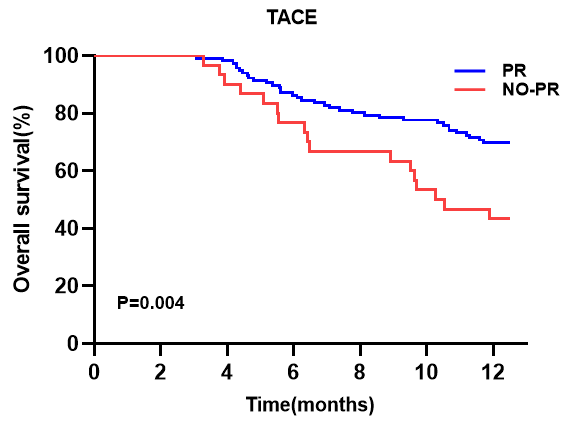

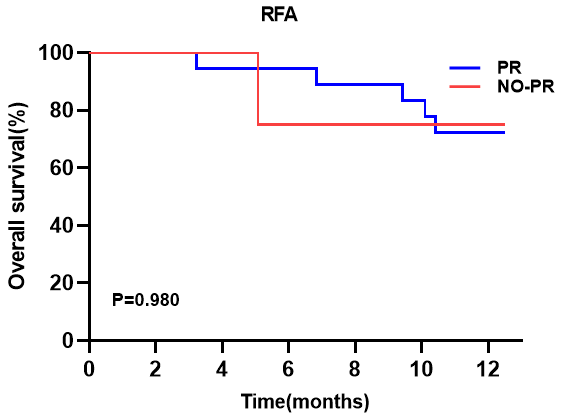


E


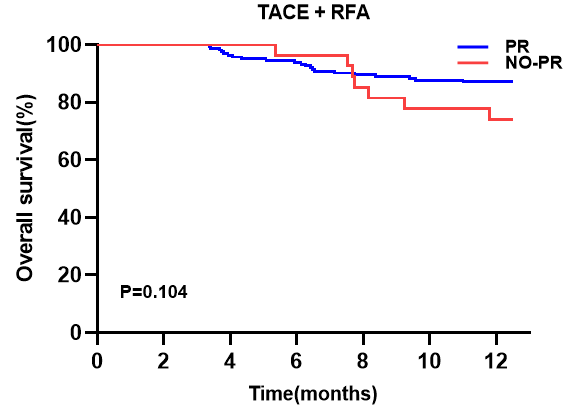


**Supplementary Figure 1** Kaplan-Meier curve showing 1-year overall survival (OS) in the two groups. **A** Patients after surgery. **B** Patients with systemic therapy . **C** Patients after TACE. **D** Patients after RFA. **E** Patients with TACE and RFA.

**Supplementary Figure 2**


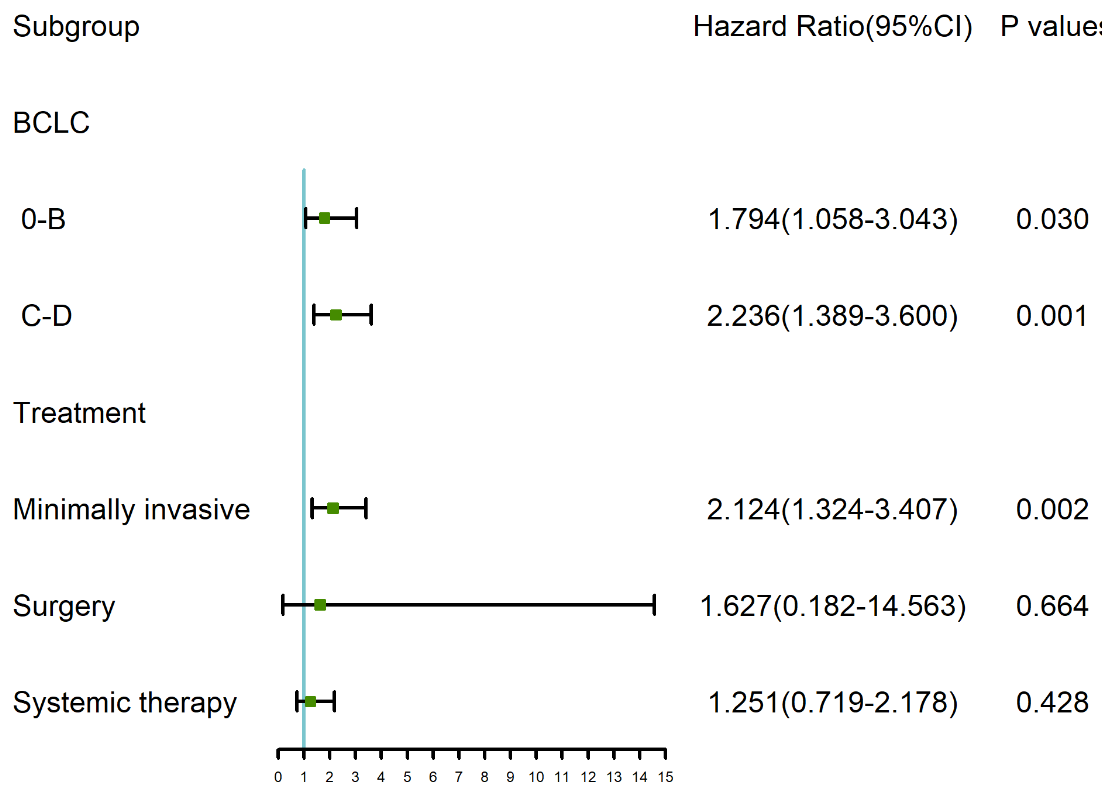


**Supplementary Figure 2** Subgroup analysis according to tumor stage and treatment modality.
